# The mediating role of public belonging in the relationship between competitive sports participation, loneliness, and self-harm among adolescents

**DOI:** 10.3389/fpsyt.2025.1695190

**Published:** 2026-01-12

**Authors:** Yadong Liang, Chunming Chen, Shangguozi Liu

**Affiliations:** 1 School of General Education, Shandong Huayu University of Technology, Dezhou, Shandong, China; 2 School of Electronic Information Engineering, Guangzhou City Polytechnic, Guangzhou, Guangdong, China; 3 Faculty of Humanities and Social Sciences, University of Nottingham Ningbo, Ningbo, Zhejiang, China; 4 School of Sports and Health, Guangdong Engineering Polytechnic, Guangzhou, China

**Keywords:** adolescent mental health, competitive sports, loneliness, self-harming behavior, sense of belonging, social identity theory

## Abstract

**Introduction:**

Loneliness and self-harming behaviors among adolescents are central concerns in global public health and education. While sports participation is known to promote well-being, the underlying mechanisms remain underexplored. Drawing on social identity theory, this study investigates whether participation in competitive sports enhances adolescents’ sense of public belonging, thereby reducing loneliness and self-harm.

**Methods:**

A quasi-experimental study was conducted with 90 middle school students randomly assigned to intervention or control groups. Over six weeks, the intervention group participated in team-based competitive sports (football, basketball, volleyball), while the control group continued regular physical education. Measures included the Psychological Sense of School Membership (PSSM), UCLA Loneliness Scale (ULS), and Self-Harm Behavior Questionnaire (SHBQ). Data were analyzed using independent-sample t-tests, structural equation modeling (SEM), and mediation analysis.

**Results:**

Competitive sports significantly increased students’ sense of public belonging. Belonging was associated with reduced self-harm and lower loneliness scores. However, within SEM, the belonging–loneliness path was only marginally significant. Overall model fit indices were weak, suggesting more complex underlying processes.

**Conclusions:**

Findings indicate that competitive sports reduce adolescents’ risk of self-harm primarily by enhancing their sense of public belonging. Evidence for buffering loneliness was weaker, implying that loneliness is shaped more directly by family, peers, and broader resources. Theoretically, this extends social identity and social capital frameworks in sport psychology. Practically, it cautions that focusing solely on performance may cause schools to overlook sports’ social benefits, limiting long-term effectiveness. Promoting adolescent mental health will require integrating school-based sports with family support, community engagement, and psychological services to build a more sustainable system of care.

## 1 Introduction

In recent years, adolescent mental health has become a central concern in both global public health and education. The widespread prevalence of loneliness and self-harm among adolescents threatens individual development and poses significant challenges to social well-being. Prior research has shown that experiences of loneliness during adolescence may carry long-term consequences into adulthood, including heightened psychological vulnerability and cumulative risks to physical and mental health (1). These challenges are further exacerbated by the rapid changes in modern social environments. While social media offers adolescents new opportunities for identity exploration and self-expression, it may also foster confusion in self-concept and encourage negative social comparisons, thereby exacerbating feelings of loneliness and emotional distress (2, 3).

Sports have been recognized as a potentially protective pathway in addressing these challenges. Systematic reviews suggest that participation in sports not only promotes adolescents’ psychological well-being and social adjustment but may also mitigate some of the negative consequences of social isolation (4). Nevertheless, not all adolescents benefit equally; factors such as psychological resilience and levels of intrinsic motivation can shape the effectiveness of sport-based interventions (5). Together, these findings highlight the vulnerability of adolescent mental health and the complex interplay of sociocultural factors, underscoring the need for practical and sustainable intervention strategies.

In efforts to find effective interventions for adolescent mental health, physical activity has been recognized as an affordable and scalable option. Evidence suggests that participating in sports improves physical health and alleviates psychosocial challenges associated with social isolation. For example, a study across 52 low- and middle-income countries found a strong link between sedentary behavior and loneliness, while active participation in sports seemed to reduce this risk (6). From a mental health perspective, the benefits of sports for adolescents extend beyond physical fitness; the very process of participation can serve as a form of psychological intervention, fostering positive emotional experiences and social connections (7).

Theoretical research further supports the psychological role of sports. Social cognitive theory posits that adolescents’ behavior and persistence are influenced by self-efficacy, social support, and collective identity (8). In this context, Competitive sports, involving cooperation, competition, and group goals, are particularly effective in building a sense of belonging and identity. Empirical studies show that participation in competitive sports significantly enhances self-concept and social identity development, effects that are evident even among populations with disabilities (9). Moreover, Research on socially vulnerable adolescents indicates that structured sports participation plays a key role in promoting mental health, not just by improving physical health but also by providing supportive group environments that help youth manage life stressorss (10).

## 2 Literature review

### 2.1 Global adolescent loneliness and the role of sports as a social context

Loneliness has become a widespread and pressing challenge for adolescents across the globe, transcending national and cultural boundaries and exerting significant influence on social and emotional development (11). In this context, the relational opportunities embedded in sports participation hold particular relevance. Sports are not limited to improving physical health; rather, they provide meaningful social experiences that can mitigate isolation and promote psychological well-being. Public belonging, defined as the sense of being recognized, valued, and accepted by one’s social environment, has been consistently identified as a protective factor against loneliness and maladaptive behaviors (12). International research demonstrates that feelings of isolation among adolescents are highly prevalent and yield similar negative outcomes across diverse cultural settings, underscoring the universal importance of fostering meaningful social connections. Participation in sports has been shown to create shared experiences that strengthen group identity, facilitate peer bonding, and promote emotional resilience (13, 14). However, the benefits of sports are not uniform, and their effectiveness depends on relational quality and program context. Competitive sports, when supported by positive team dynamics, can foster developmental pathways that reinforce identity and collective goals (15), though negative encounters, performance pressures, or injuries may diminish these benefits and compromise psychological health (16, 17). In sum, sports participation may provide a promising avenue to address adolescent loneliness, but its positive impact requires supportive environments and meaningful social interactions.

### 2.2 Public belonging as a protective social resource for youth well-being

Beyond the sports domain, a strong sense of belonging continues to function as a foundational protective factor in adolescents’ broader social environments. Young people who lack social inclusion and meaningful interpersonal connections are more vulnerable to experiencing persistent loneliness and identity instability, including in digital settings where social comparisons and exclusion can amplify emotional distress (18). Conversely, supportive peer relationships and nurturing environments contribute to healthier psychological development and pro-social functioning (19). From a developmental perspective, organized sports have historically been recognized as important contexts for socialization, providing unique opportunities for adolescents to cultivate cooperation, responsibility, and emotional regulation (7, 20). Yet, the relationship between sports participation and mental health is not simply linear; rather, belonging serves as a critical mechanism through which social experiences in sports translate into improved well-being, reducing the risk of loneliness and self-harm. This aligns with growing recognition that emotional resilience in youth is shaped not only by individual traits but also by relational and contextual resources. In contemporary settings characterized by heightened academic pressures and digital exposure, the need for accessible, community-based structures that strengthen belonging and emotional security has become increasingly urgent. Taken together, public belonging emerges as a key explanatory factor linking sports participation to adolescent mental health outcomes, reinforcing the importance of social connectedness in mitigating loneliness and reducing self-harm vulnerabilities.

### 2.3 Theoretical foundations: identity, social capital, and stress vulnerability

The present study is grounded in three complementary theoretical frameworks that together provide a robust foundation for understanding how competitive sports participation may influence adolescent mental health outcomes. Social identity theory (21) posits that individuals develop a sense of self and belonging through identification with social groups; within team-based sports, adolescents reinforce their identity and gain social recognition through shared goals and collective experience. Social capital theory (20, 22) emphasizes the value of social networks, trust, and reciprocal support, suggesting that sports teams may serve as micro-communities where youth can access emotional and relational resources essential for social development. Additionally, the stress–vulnerability model (20) highlights that self-harm is more likely when stress exceeds coping resources, implying that belonging and supportive peer interactions fostered in sports may buffer emotional strain. Although extensive literature supports the developmental benefits of physical activity (23–25), empirical studies examining the specific mechanisms in competitive school sports remain limited, particularly regarding cross-cultural differences and potential moderating factors such as individual perfectionism or socio-environmental constraints (26, 27). Therefore, this study advances current knowledge by empirically testing a theoretical pathway model linking competitive sports involvement, public belonging, and adolescent mental health, offering implications for both developmental psychology and school-based mental health interventions.

## 3 Methods

### 3.1 Research objectives

This study aims to examine whether short-term engagement in competitive team sports can enhance adolescents’ sense of belonging within their peer group, and in turn, reduce feelings of loneliness and tendencies toward self-harming behavior. By focusing on the psychological mechanism of public belonging, the study seeks to clarify how structured sports interventions may promote mental well-being among adolescents.

### 3.2 Hypotheses

H1: Students who participate in competitive team sports are expected to report significantly higher levels of perceived public belonging compared to their peers in the control group.

H2: Elevated levels of perceived public belonging are anticipated to be negatively associated with loneliness and self-harming tendencies.

H3: Perceived public belonging is hypothesized to mediate the relationship between participation in competitive team sports and students’ loneliness and self-harming tendencies.

### 3.3 Overview of the experimental design

To cite **Table 1**, which summarizes the overall experimental design, including study type, sample allocation, intervention duration, and measurement time points.

**Table 1:** Overview of the experimental design.

Item	Description
Design Type	Quasi-experimental design (Pre–Post Test with Control Group)
Sample Size	Approximately 90 participants, randomly assigned to two groups (intervention vs. control)
Unit of Assignment	Class groups or interest-based teams, to avoid treatment contamination
Grouping Method	Natural class allocation with efforts to control for baseline differences
Intervention Duration	6 weeks, with 3 sessions per week, each lasting 60 minutes
Measurement Time Points	T0 (pretest/baseline) and T1 (posttest, including all dependent variables)
Data Structure	Individual level × time (repeated measures), suitable for path analysis and mediation testing

### 3.4 Intervention and control conditions

To cite **Table 2**, detailing the structure of the intervention and control conditions and the corresponding indicator system framework.

**Table 2:** Indicator system framework.

Primary dimension	Secondary indicator	Theoretical basis	Data source & instrument
Competitive Sports Participation	Participation Grouping (Intervention vs. Control)	Sport Socialization Theory: Competitive sports promote social identity construction and group affiliation (Carron & Hausenblas, 1998)	Experimental grouping (0 = control group, 1 = intervention group); researcher records
	Participation Intensity (Attendance Rate, Engagement Level)	Behavioral Activation Theory: Higher attendance rates are positively associated with intervention outcomes (Bandura, 1997)	Attendance logs and match participation records during intervention period
Public Sense of Belonging	School Belonging	Psychological Need Satisfaction Theory: A sense of belonging reflects fundamental psychological needs (Baumeister & Leary, 1995)	PSSM – Psychological Sense of School Membership Scale (8-item version)
	Public Visibility/Being Seen	Social Identity Theory (Tajfel & Turner, 1986): Group identity is reinforced by public recognition	SCI-2 – Social Connectedness Index (subscale: public visibility dimension) + custom visibility perception questionnaire
Mental Health	Loneliness	Social Isolation Theory (Cacioppo & Hawkley, 2009): Loneliness results from perceived disconnection from others	UCLA Loneliness Scale (ULS-8)
	Self-Harm Tendency	Stress–Vulnerability Model (Linehan, 1993): Risk of self-harm increases under cumulative psychosocial stress	Self-Harm Behavior Questionnaire (SHBQ short form) + ASQ screener (used only for pre-screening, not included in path model)

(1) Intervention Group: Team-based Competitive Sports.

Participants engaged in a structured school-based sports program modeled on small-scale campus “leagues” or “challenge matches,” in which each student selected one sport from football, basketball, or volleyball. The intervention lasted six weeks, with three 60-minute sessions per week, and each session combined technical skill training with formalized team competition. The program adopted an organized group-based format supported by a points leaderboard and corresponding reward mechanisms to sustain motivation and engagement. To cultivate a sense of public recognition and shared identity, results were publicly announced through bulletin board postings, campus broadcasts, and end-of-series award presentations. Throughout the program, emphasis was placed on fostering collaboration, mutual encouragement, and collective honor rather than prioritizing individual performance or personal achievements, thereby reinforcing a socially supportive climate aligned with the developmental goals of belonging and team-based identity building.

(2) Control Group: General Physical Activities.

Participants engaged in standard physical education activities, including fitness training, rope skipping, rhythmic exercises, and light recreational games. The total activity time matched that of the intervention group, but without any team-based competition, group confrontation, or public display elements.

### 3.5 Indicator system

To cite **Table 3**, which presents the indicator system, theoretical foundations, and measurement instruments for all core variables.

**Table 3:** Variable definitions and measurement.

Variable type	Variable name	Operational definition	Measurement method & instrument	Theoretical framework
Independent Variable	Competitive Sports Participation	Whether the participant engaged in a 6-week competitive team sports intervention (football, basketball, or volleyball), 3 times per week, 60 minutes per session	Grouping variable: intervention group = 1, control group = 0; supplemented by attendance records and participation intensity	Sport Socialization Theory (Carron & Hausenblas, 1998)
Mediator Variable	Public Sense of Belonging	The degree to which an individual feels seen, accepted, and socially connected in public school spaces	PSSM (8-item version); SCI-2 Public Belonging Subscale; higher scores indicate stronger perceived belonging	Need to Belong Theory (Baumeister & Leary, 1995); Social Identity Theory (Tajfel & Turner, 1986)
Dependent Variables	Loneliness (primary outcome)	Subjective experience of social disconnection and lack of emotional closeness	UCLA Loneliness Scale (ULS-8); higher scores reflect greater loneliness	Social Isolation Theory (Cacioppo & Hawkley, 2009)
	Self-Harm Tendency (secondary outcome)	Individual’s expressed intention or likelihood of engaging in self-injury when facing psychological stress or emotional pain	Self-Harm Behavior Questionnaire (SHBQ, short form); ASQ screener (used only for initial screening, not included in inferential analysis)	Stress–Vulnerability Model (Linehan, 1993)

### 3.6 Variable descriptions

To cite **Table 4**, reporting the operational definitions and descriptive statistics of the key variables.

**Table 4:** Descriptive statistics of key variables.

Variable	N	Mean	Std. dev.	Min	25th percentile	Median	75th percentile	Max
Public Belonging (T0)	90	1.94	0.84	1	1	2	3	3
Public Belonging (T1)	90	2.74	1.09	1	2	3	4	5
Loneliness (T0)	90	0.93	0.87	0	0	1	2	3
Loneliness (T1)	90	1.5	1	0	1	2	2	4
Self-Harm Behavior (T0)	90	2.92	0.88	1	2	3	3	4
Self-Harm Behavior (T1)	90	2.38	1.02	1	2	2	3	4

## 4 Empirical results

### 4.1 Descriptive statistics

**Figure 1:**
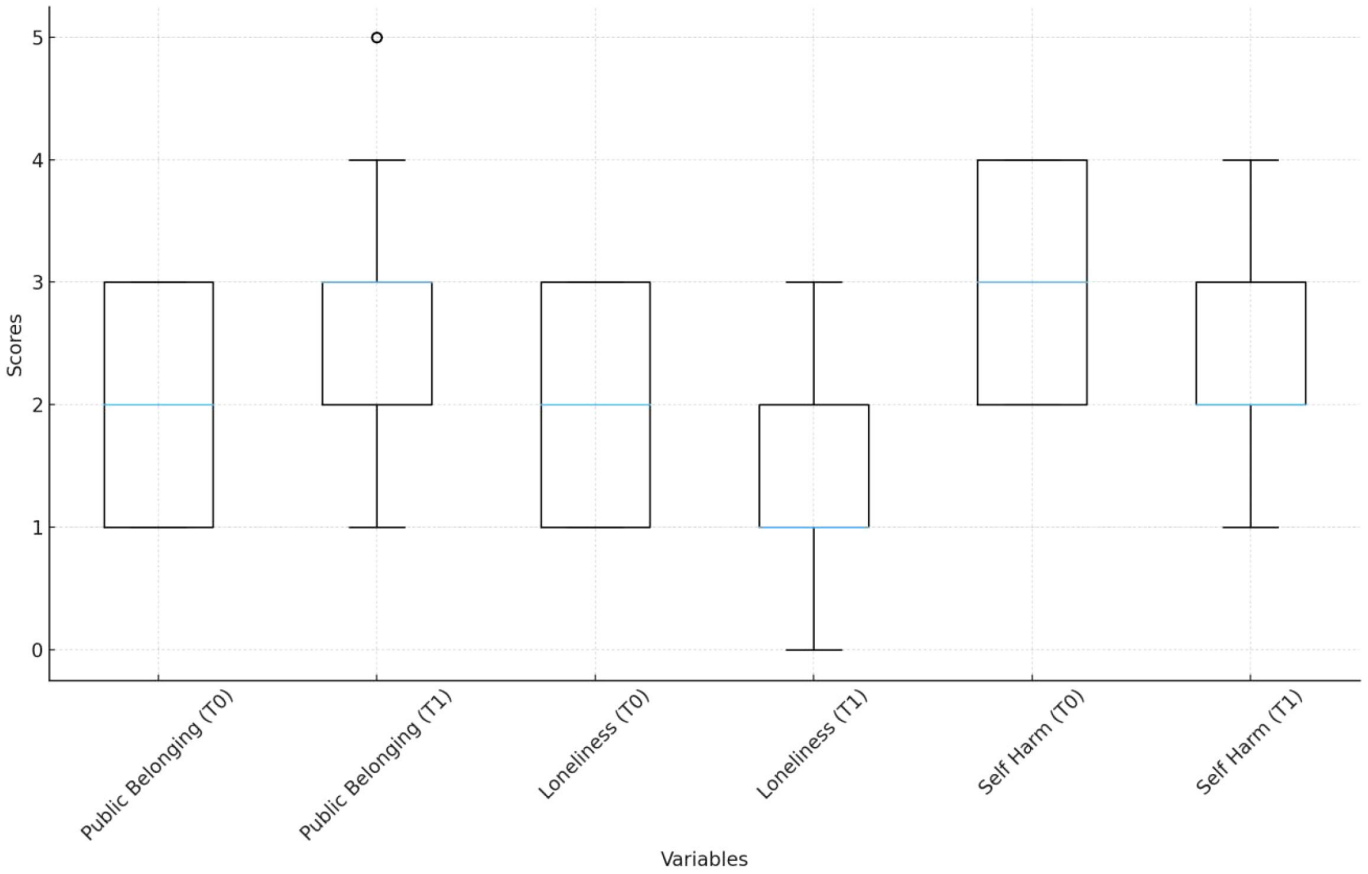
Descriptive statistics for key variables.

As shown in **Tables 5**, and **6**, and **Figure 1**, students in the intervention group showed noticeable improvements after participating in the competitive sports program. Their sense of public belonging rose from 1.94 in the pre-test to 2.74 in the post-test. At the same time, their reported feelings of loneliness dropped from 1.93 to 1.50, and self-harming behaviors decreased from 2.92 to 2.38. These patterns indicate a generally positive shift in psychological well-being associated with the intervention.The average attendance rate was 70.1% (SD = 6.2%), meaning most students took part in about 70% of the sessions, although participation varied. This suggests that the sports-based intervention helped build a stronger sense of group identity, eased feelings of isolation, and may have helped reduce self-harm risks. However, not all students benefitted equally.The decline in loneliness, for instance, while statistically significant, was relatively modest. This hints that the causes of loneliness among students are multifaceted and cannot be addressed by physical activity alone. Variation in attendance rates is also important—students with higher attendance showed greater improvement, while those with lower attendance showed smaller changes.All of this points to a key insight: the success of these interventions depends not only on the activities themselves but also on structural factors like institutional support and equitable access to resources. Without these, even a well-designed program may struggle to achieve lasting or widespread impact.

**Table 5:** Descriptive statistics of attendance rates in intervention and control groups.

Group	N	Mean attendance rate (%)	Standard deviation (SD %)
Control (0)	41	69.54%	5.43%
Intervention (1)	49	70.08%	6.17%

**Table 6:** Results of validity and reliability analysis.

Scale	Cronbach’s α
Public Belonging (PSSM)	0.78
Loneliness (ULS)	0.92
Self-Harm Behavior (SHBQ)	0.92

### 4.2 Validity and reliability testing

**Figure 2:**
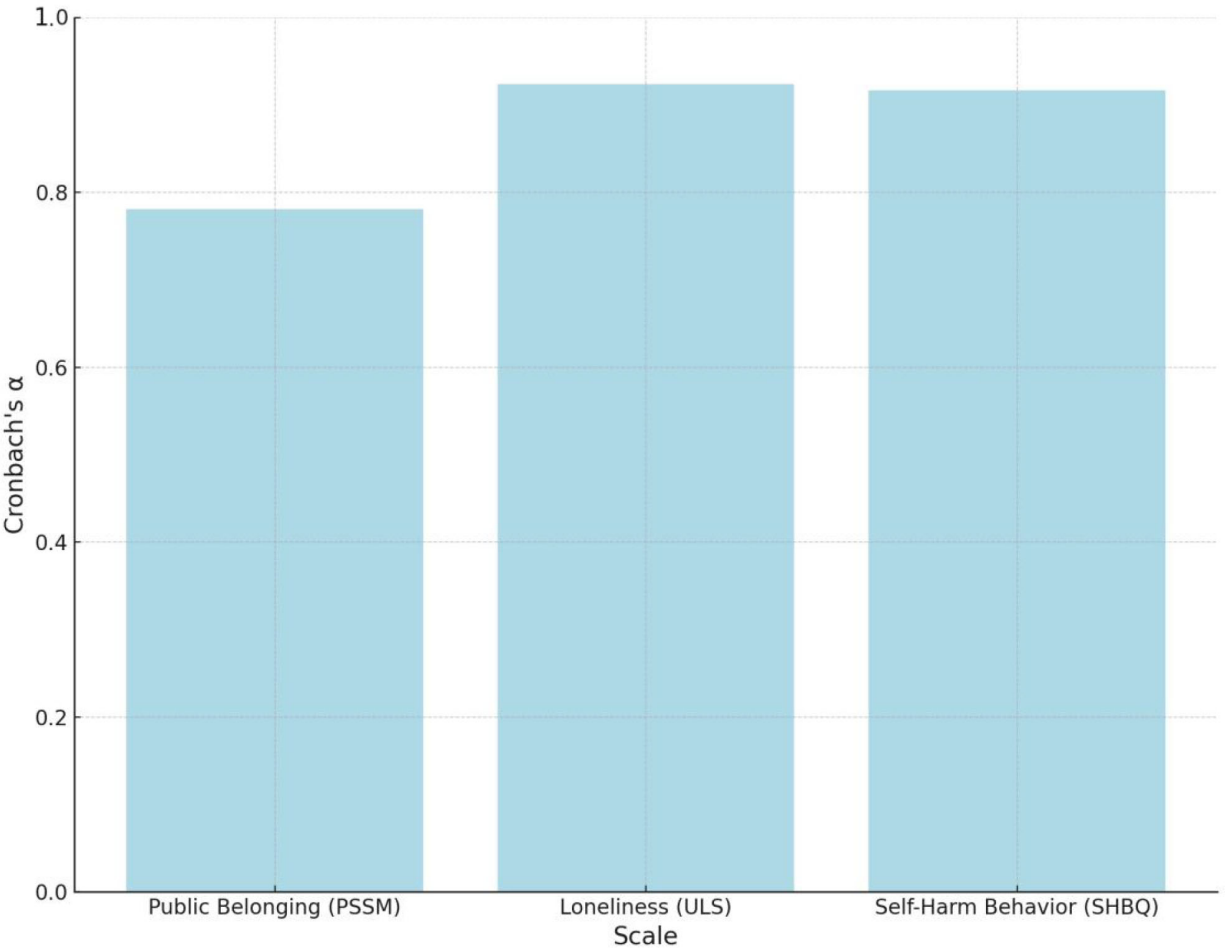
Cronbach’s alpha for different scales.

As shown in **Table 6** and **Figure 2**, the Cronbach’s alpha coefficients for the PSSM, ULS, and SHBQ scales were 0.78, 0.92, and 0.92, respectively. In general, a Cronbach’s alpha above 0.70 is considered acceptable, while values above 0.80 indicate good internal consistency. The alpha coefficient for the PSSM scale (α = 0.78) indicates an acceptable level of reliability, albeit slightly below the conventional threshold for strong consistency. Given that school belonging is inherently a multidimensional construct, future studies might consider expanding the scale items or refining the measurement approach to enhance its reliability. In contrast, The alpha values for the ULS and SHBQ (both α = 0.92) indicate excellent internal consistency, suggesting that these scales yielded highly stable results in this sample. However, it is important to note that such high reliability coefficients may also reflect item redundancy or limited construct differentiation. Overly homogeneous scales may miss the nuanced expressions of loneliness and self-harm behaviors, particularly in diverse socio-cultural settings. From a critical standpoint, while these reliability results provide a solid psychometric foundation for the study’s analyses, they also raise questions about the extent to which these instruments fully capture the psychological experiences of adolescents across different cultural or demographic groups. For instance, adolescents from varying socio-cultural or familial backgrounds may interpret or respond to items about loneliness, belonging, or self-harm in markedly different ways. Therefore, future research should not only examine the cross-group validity of these measures but also consider incorporating mixed-method approaches or culturally responsive adaptations to ensure broader interpretability and ecological validity.

### 4.3 Independent samples t-test

**Figure 3:**
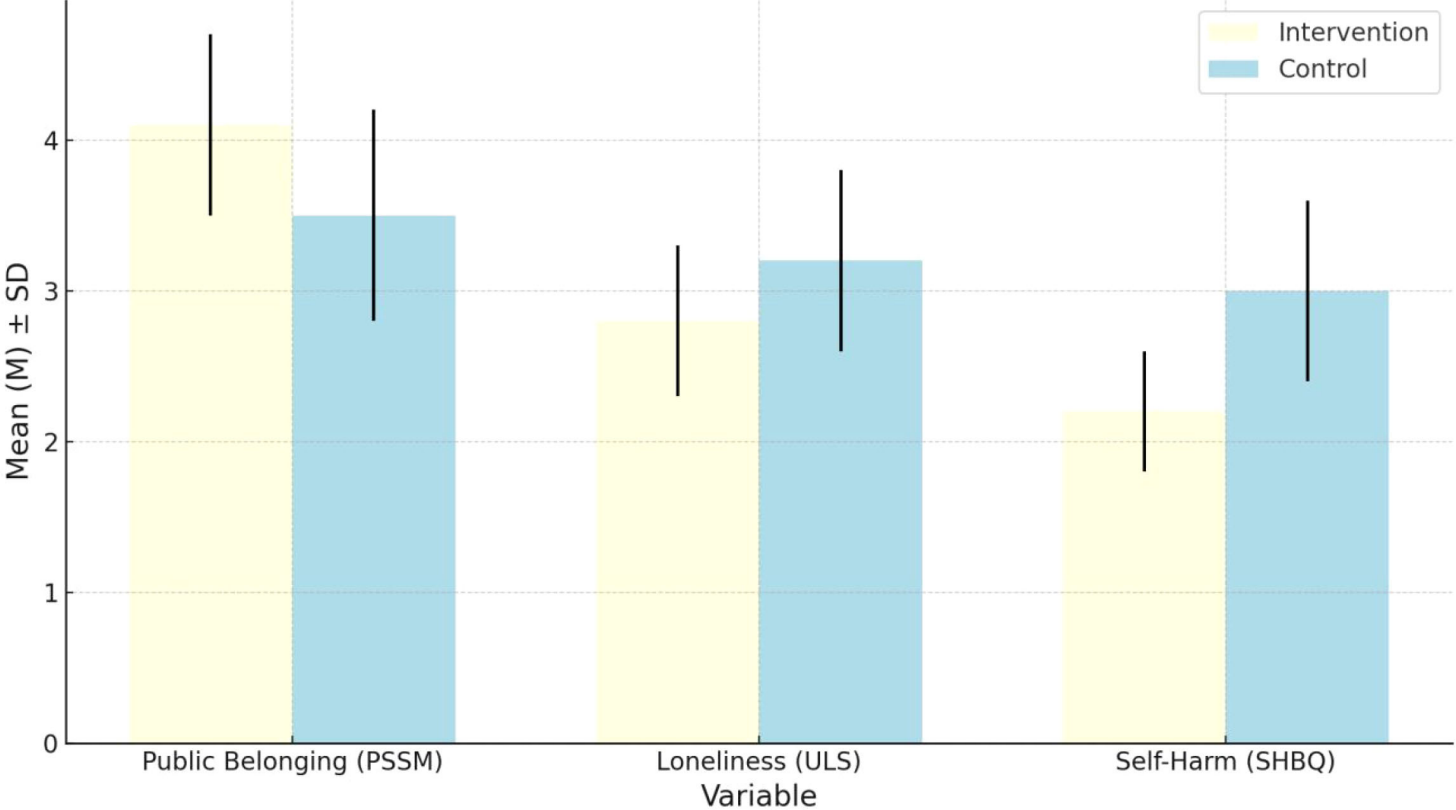
Independent samples t-test results (intervention vs control).

**Table 7** and **Figure 3** show that students who participated in competitive sports reported significantly higher levels of public sense of belonging (PSSM) than those in the control group (t = 5.51, p <0.001), with mean scores of 4.1 and 3.5 respectively. This finding suggests that engaging in team-based sports can help adolescents feel more connected to their social environment. However, the effect may not be equally strong across different groups. Factors such as school location, access to resources, and students’ family background may influence how effective the intervention is. It reminds us that psychological benefits are not just shaped by the activity itself, but by the conditions under which it takes place. Regarding loneliness (ULS), although the intervention group showed a lower average score (2.8) than the control group (3.2), the difference was not statistically significant (t = 1.39, p =0.169). This indicates that sports participation alone might not be sufficient to reduce feelings of loneliness among adolescents. Loneliness is often tied to deeper issues, such as the lack of emotional support from family, poor peer relationships, or low social confidence. For some students, sports may offer brief relief but are unlikely to address the root causes. Future programs should consider combining physical activities with psychological counseling and peer support strategies. In terms of self-harm behavior (SHBQ), the intervention group scored significantly lower than the control group (t = –7.35, p < 0.001), suggesting that regular participation in structured sports can help reduce harmful behaviors. This may be because of improved emotional regulation and stronger peer bonds formed during group activities. Still, given the complexity of self-harm, sports should not be seen as a stand-alone solution. Students facing serious psychological challenges may require long-term support, including therapy, family involvement, and school-based mental health services. The results show promise, but also highlight the importance of a more integrated approach.

**Table 7:** Results of the independent samples t-test.

Variable	Intervention (M ± SD)	Control (M ± SD)	t-value	p-value	Significance
Public Belonging (PSSM)	4.1 ± 0.6	3.5 ± 0.7	5.51	< 0.001	***
Loneliness (ULS)	2.8 ± 0.5	3.2 ± 0.6	1.39	0.169	-
Self-Harm (SHBQ)	2.2 ± 0.4	3.0 ± 0.6	-7.35	< 0.001	***

*p < 0.05; **p < 0.01; ***p < 0.001.

### 4.4 Paired samples t-test

**Figure 4:**
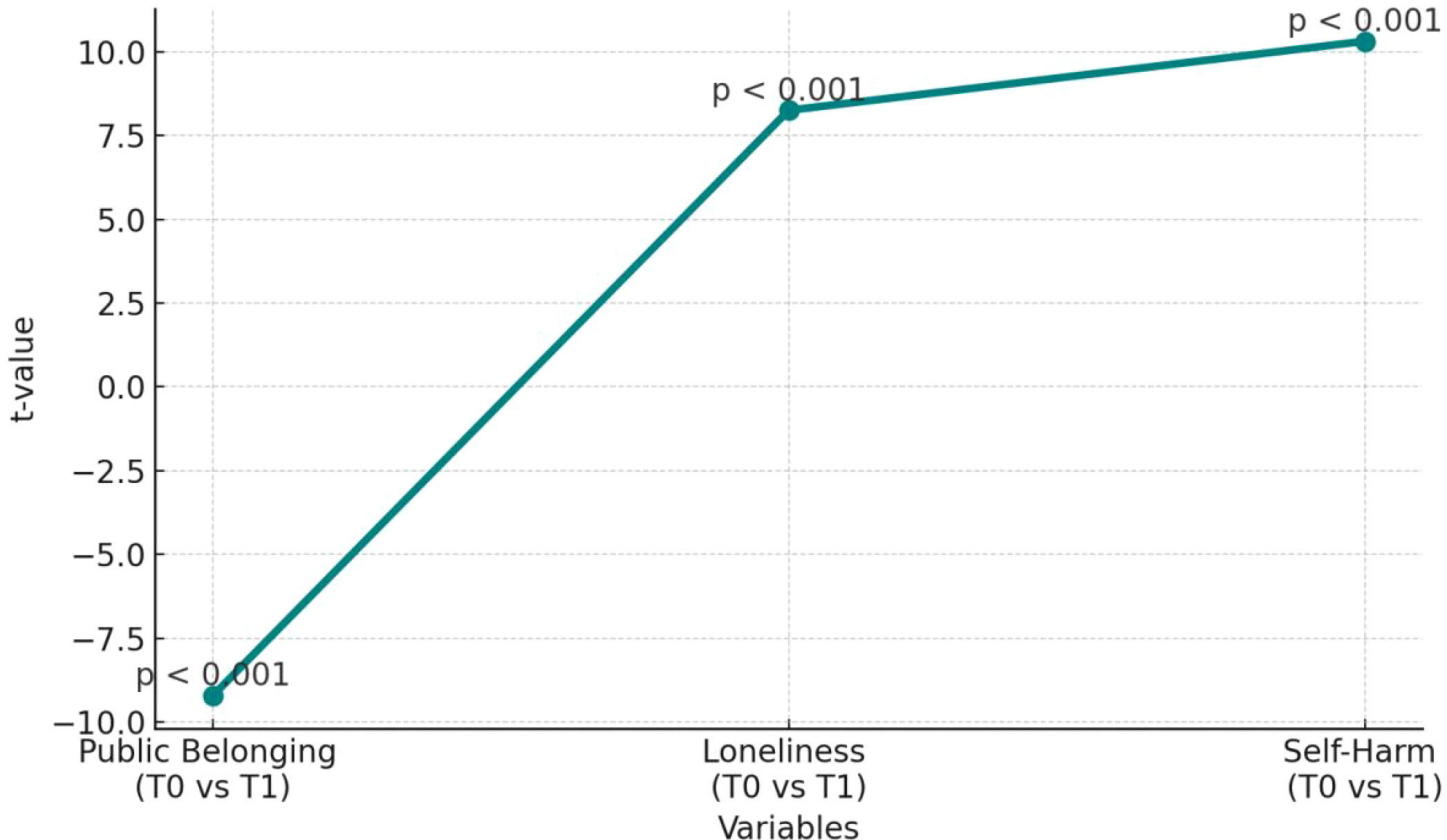
Paired sample T-test results.

As shown in **Table 8** and **Figure 4**, the paired samples t-test for Public Sense of School Membership (PSSM) revealed a significant increase after the intervention (t = –9.21, p < 0.001), with mean scores rising from 1.94 (T0) to 2.74 (T1). This indicates that participation in competitive sports effectively enhanced adolescents’ sense of belonging in the school context. From a critical perspective, this finding highlights the potential of team-based activities in fostering social interaction and collective identity among youth. However, the observed effect may not be consistent across all individuals. In particular, students from different socioeconomic backgrounds may benefit to varying degrees. Future studies should explore how to mitigate such disparities to ensure equitable access and impact of similar interventions. For loneliness (ULS), the paired t-test also yielded significant results (t = 8.25, p < 0.001), with mean scores dropping from 1.93 at T0 to 1.50 at T1. While this supports the idea that physical activity can help alleviate feelings of loneliness, attention must be given to the heterogeneity of outcomes. Especially for individuals experiencing chronic loneliness or lacking social skills, a single intervention based on sports may prove insufficient. The root causes of loneliness are often multifaceted, involving family support, emotional resilience, and personality traits. Therefore, although the data confirms a general reduction in loneliness, it also underscores the limitations of stand-alone interventions. Future programs should incorporate broader mental health support services to achieve sustained and individualized improvements. As for self-harming behavior (SHBQ), the pre–post comparison revealed a significant decrease (t = 10.31, p < 0.001), with average scores dropping from 2.92 to 2.38. This suggests that competitive sports may help reduce self-harm tendencies, likely through improvements in emotional regulation and social connectedness. Nonetheless, from a critical standpoint, self-harming behavior remains a complex issue. While physical activity may offer short-term emotional benefits, its effects may not extend equally to all adolescents—particularly those with more severe psychological difficulties. Thus, even though the intervention demonstrated significant effectiveness, but it should not be regarded as a one-size-fits-all solution. For at-risk individuals, a comprehensive approach that includes sports, psychological counseling, and family engagement is likely necessary. Future interventions should consider these intersecting factors rather than relying solely on athletic participation.

**Table 8:** Results of paired samples t-test.

Variable	t-value	p-value	Significance
Public Belonging (T0 vs T1)	-9.21	< 0.001	***
Loneliness (T0 vs T1)	8.25	< 0.001	***
Self-Harm (T0 vs T1)	10.31	< 0.001	***

*p < 0.05; **p < 0.01; ***p < 0.001.

### 4.5 Structural equation modeling

**Figure 5:**
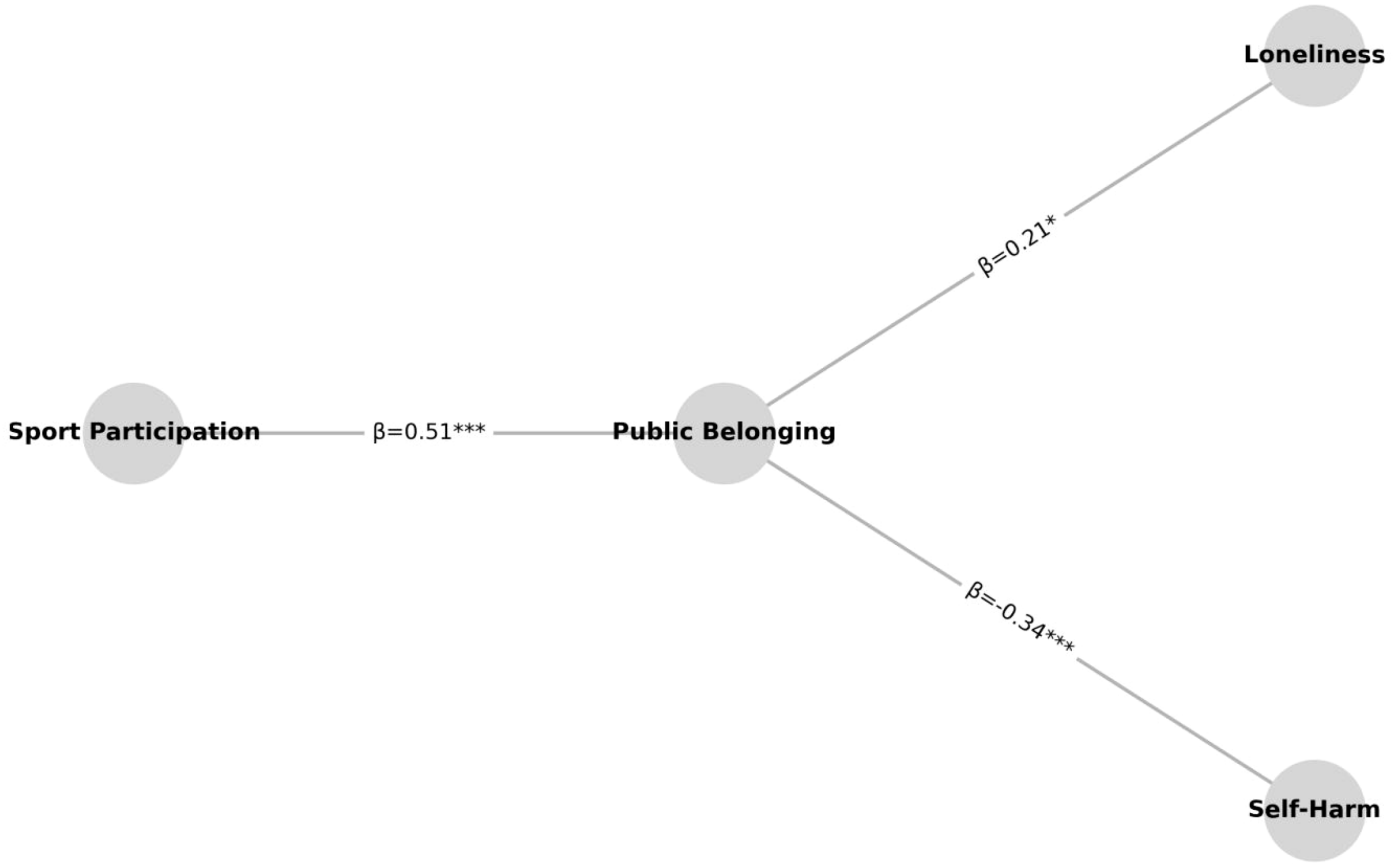
Structural equation model (SEM) path diagram.

As shown in **Table 9** and **Figure 5**, the competitive sports intervention significantly improved adolescents’ sense of school belonging (β = 0.51, p < 0.001). This suggests that sports can play a much more meaningful role than just physical exercise—it can help young people feel more connected, included, and emotionally supported in their school community. In other words, sports can build a sense of identity and belonging, which is essential for mental health, especially in school settings. But looking at this critically, such positive effects might not apply equally to all groups. Many schools still treat sports mainly as a way to train top athletes and focus on performance rather than participation or emotional support. As a result, the full potential of sports as a psychological intervention often goes unnoticed or underused. If schools continue to emphasize competition over connection, they may miss an opportunity to improve students’ mental well-being in a more inclusive and sustainable way. The model indicates that a stronger sense of belonging significantly reduces self-harm behaviors (β = −0.34, p < 0.001). This confirms how important emotional connection and group support are for protecting mental health. Yet in practice, many school-based mental health efforts still focus on helping individuals after problems appear, rather than creating supportive group environments in the first place. Sports—especially team-based, inclusive programs—offer a chance to build such environments. But this only works if schools shift from seeing mental health as a personal issue to recognizing the social context that shapes it. Interestingly, the relationship between school belonging and loneliness was marginally significant (β = 0.21, p = 0.044). That means while belonging can help reduce loneliness, it’s not a guaranteed solution. Loneliness is complex. It can be shaped by family dynamics, peer relationships, cultural background, or even how much time someone spends online instead of in real-life interactions. For some students—especially those with deeper emotional needs or fewer support systems—just playing sports may not be enough. So while this study shows that sports interventions have real value, it also highlights their limits. If future interventions want to truly support adolescents’ mental health, they’ll need to do more than organize activities—they’ll need to connect those activities to broader systems of family, peer, and community support. Otherwise, the benefits may be short-lived or reach only some students.

**Table 9:** Path analysis results of the structural equation model.

Path	β	t	p	Significance
X→M (Group → PSSM)	0.51	>7.0	<.001	***
M→Y_1_ (PSSM → ULS)	0.21	2	0.044	*
M→Y_2_ (PSSM → SHBQ)	-0.34	-3.5	<.001	***

*p < 0.05; **p < 0.01; ***p < 0.001.

### 4.6 Mediation effect model

**Table 10** presents the results of the mediation analysis. A significant indirect effect was found in the pathway from participation in competitive sports to self-harming behaviors through perceived public sense of belonging (indirect effect = −0.174, 95% CI [−0.297, −0.061]). This suggests that sports-based interventions help reduce adolescents’ risk of self-injury by enhancing their sense of connection and belonging. The robustness of this effect highlights the essential role of social affiliation as a psychological safeguard in youth mental health. However, the mediating role of public sense of belonging in the pathway from sports participation to loneliness was marginally significant (indirect effect = 0.108, 95% CI [−0.002, 0.209]). This result requires cautious interpretation, as the confidence interval borders on non-significance, indicating that the effect may not be stable or generalizable across populations. While group belonging may contribute to reducing loneliness, it appears insufficient on its own. Loneliness is often shaped by more complex and layered factors, including the absence of family support, peer rejection, unequal access to educational resources across urban and rural areas, and increased reliance on digital forms of social interaction. Critically speaking, when schools or policymakers rely solely on group belonging as the intervention mechanism, they risk framing loneliness as an individual failing rather than a social issue. This could inadvertently shift the burden onto students, obscuring the institutional and structural roots of emotional distress. In reality, adolescents from disadvantaged backgrounds—those lacking family support or community infrastructure—are often the least likely to benefit from sports interventions in a sustainable way. Without complementary strategies like family counseling, peer mentoring, and embedded community resources, sports alone may fall short in addressing deeper emotional vulnerabilities. Overreliance on school-based sports as a “catch-all” solution may, paradoxically, reinforce the very inequalities it aims to address.

**Table 10:** Results of the mediation effect model.

Indirect path	Estimate	95% CI	Significance
X → M → ULS	0.108	[−0.002, 0.209]	Marginally significant
X → M → SHBQ	−0.174	[−0.297, −0.061]	Significant

## 5 Discussion

### 5.1 Psychological benefits of sport are not automatic: tensions between emotional support mechanisms and structural constraints

This study found that students who participated in the intervention experienced a substantial increase in their sense of public belonging, rising from 1.94 to 2.74 (t = –9.21, p <.001), which in turn contributed to reductions in self-harm (β = –0.34; indirect effect = –0.174, 95% CI [–0.297, 0.061]). These findings illustrate that sport can function as a relational space where students feel noticed, valued, and connected. This pattern is consistent with prior evidence showing that social identity processes in sport contexts heighten susceptibility to peer norms and group influence, thereby shaping emotional and behavioral outcomes among young athletes (28).

At the same time, the average attendance rate of 70.1%, with notable variation across students, signals that psychological gains were closely tied to consistent participation. Informal student feedback also suggested that academic pressure, physical fatigue, and household caregiving responsibilities sometimes limited attendance. These patterns reveal that the psychological benefits of sport do not emerge simply by offering a program or securing short-term participation. Instead, emotional gains appear to depend on sustained engagement and environments where students are emotionally held by instructors and peers. This finding aligns with systematic reviews and meta-analyses demonstrating that sport participation is most beneficial when embedded in supportive, structured contexts rather than treated as an isolated activity (29–31). Sport-based programs therefore need to be framed not only as athletic opportunities but also as guided emotional spaces that require relational work and consistent support.

### 5.2 Inequalities on the playing field: participation gaps, family resources, and disparities in psychological support

Although self-harm scores declined meaningfully among participants (from 2.92 to 2.38), the intervention’s effects on loneliness were more uneven. Students who maintained higher attendance tended to show clearer gains, whereas those with inconsistent attendance often demonstrated flatter trajectories or persistent loneliness. Qualitative observations suggested that such students were more likely to face external constraints, including family responsibilities and limited emotional encouragement at home. These findings highlight that sport, while offered universally, may not be accessed uniformly and may reproduce differences in emotional opportunity. Prior longitudinal research has similarly shown that the developmental benefits of sport are moderated by family resources, parental support, and broader social conditions rather than participation alone (32, 33). Adolescents with stable support systems were able to remain involved and benefit more, whereas those already facing social or familial strain struggled to sustain participation. The risk is that school sport programs, when assumed to be inherently equalizing, may unintentionally privilege students who already possess social capital and the emotional resources to persist. This concern echoes broader critiques in youth sport research, which caution that performance-oriented cultures may reinforce health-compromising behaviors and psychosocial inequalities if supportive structures are absent (34, 35). Effective implementation therefore requires recognizing that psychological benefits are distributed through channels shaped by time availability, parental support, and emotional bandwidth, not solely by motivation or ability.

### 5.3 Beyond the sport program: integrating family, school, and community to build a supportive socio-emotional ecology

The effects on loneliness were modest and the structural model fit was limited (CFI = 0.565; RMSEA = 0.334), indicating that sport alone may not fully address the complex emotional challenges adolescents face. Several students reported feeling connected during sessions yet lonely once they returned home, suggesting that the protective function of sport, while meaningful, is fragile without reinforcement in other relational settings. This finding is consistent with evidence showing that loneliness in youth is shaped by long-term interpersonal trajectories and broader social ecologies that extend beyond school-based activities (29, 30, 36). For young people who lack consistent emotional availability from caregivers or who experience fragile peer ties, school-based sport may provide temporary relief but not durable change. These findings point to the need for multi-layered support extending beyond the athletic field. International position statements and methodological research in sport psychology further emphasize that sustainable mental health promotion requires coordinated systems involving families, schools, and communities, alongside theoretically grounded and adequately powered research designs (35, 37, 38). In this context, sport should be understood as a valuable entry point into emotional support rather than a comprehensive solution. Building durable psychological resilience requires a coordinated system of care that accompanies adolescents beyond scheduled practice and into their everyday relational environments.

## 6 Conclusion

This study, situated in the everyday realities of a school environment, demonstrates that organized competitive sport can serve as a meaningful context for adolescents to cultivate a shared sense of belonging and emotional groundedness. While the intervention yielded measurable benefits in reducing self-harm through enhanced belonging, its broader value lies in offering students a reliable setting for connection, recognition, and shared purpose—experiences that are increasingly scarce in achievement-driven school cultures. The findings suggest that sport functions most effectively not as a standalone remedy but as one pathway through which schools can strengthen the relational fabric that supports young people’s emotional development. In this sense, sport is less about isolated episodes of activity and more about creating rhythms of participation, mutual responsibility, and encouragement that accompany students through the uncertainties of adolescence. Looking ahead, the significance of this work rests in showing that even modest, practice-integrated programs can open accessible routes to emotional support when schools remain attentive to continuity, inclusion, and the everyday textures of student experience. Sustaining such efforts will require not only physical participation but also pedagogical intention, institutional patience, and collaboration beyond the gymnasium, so that sport becomes a steady source of connection rather than an occasional intervention.

## 7 Limitation and future

The present study was conducted in a natural school setting, and several contextual constraints merit attention. The sample size was relatively modest (N = 90) and the intervention lasted six weeks. While this design enhances ecological relevance by reflecting realistic educational conditions, it may also influence the stability of observed effects and limit the generalizability of the findings. The effect of belonging on loneliness was only marginally significant, which is consistent with the developmental characteristics of adolescence. During this period, young people are particularly sensitive to peer relations and changes in their social environment, and experiences of loneliness often fluctuate accordingly (39, 40). Meanwhile, adolescents’ sense of belonging is still developing and consolidating (41), which may contribute to variability in related effects. This pattern suggests that while sports participation can offer emotional support and social connection, sustained reductions in loneliness may require a broader support network involving familial involvement, peer support, school climate initiatives, and community resources, thereby providing a continuous and caring support system for adolescents. The average attendance rate of approximately 70% reflects the influence of academic demands, health conditions, and family responsibilities on students’ participation in school activities. Although this enhances ecological validity by capturing real-world participation, variability in attendance may attenuate the intervention effect and constrain generalizability. Future research may therefore explore dose–response relationships and potential interaction mechanisms to better understand how differing levels of participation shape psychological benefits. Additionally, the suboptimal model fit suggests that the underlying mechanisms may be more complex than the current single-mediator framework captures, possibly due to sample size, intervention duration, and theoretical model structure. Subsequent studies could expand sample diversity, extend follow-up periods, and employ multilevel or longitudinal structural equation modeling, while also examining moderators such as gender, family background, and access to social support. Cross-cultural validation would further support the robustness and applicability of these findings across different contexts.

## Data Availability

The raw data supporting the conclusions of this article will be made available by the authors, without undue reservation.

## Appendix

**Appendix 1:** Basic demographic information and variable scores of selected participants.

Participant ID	Gender	Grade Level	Attendance Rate (%)	Matches per Week	Public Belonging _T0	Public Belonging _T1	Loneliness _T0	Loneliness _T1	Self-Harm _T0	Self-Harm _T1
202501	Male	Grade 7	78	2	3	5	3	2	3	2
202502	Male	Grade 8	68	2	2	3	2	4	2	4
202503	Male	Grade 8	76	1	2	5	1	2	1	1
202504	Male	Grade 9	71	3	3	4	2	3	2	2
202505	Female	Grade 7	73	1	3	3	1	0	4	4
202506	Male	Grade 7	74	2	4	1	1	1	3	2
202507	Female	Grade 8	75	1	2	3	3	3	2	1
202508	Female	Grade 7	79	2	2	2	2	2	3	3
…	…	…	…	…	…	…	…	…	…	…
